# Anti-Inflammatory and Anti-Apoptotic Effects of Stybenpropol A on Human Umbilical Vein Endothelial Cells

**DOI:** 10.3390/ijms20215383

**Published:** 2019-10-29

**Authors:** Li Zhang, Feifei Wang, Qing Zhang, Qiuming Liang, Shumei Wang, Minghua Xian, Feng Wang

**Affiliations:** 1 Key Laboratory of Digital Quality Evaluation of Chinese Materia Medica of State Administration of TCM, School of Traditional Chinese Medicine, Guangdong Pharmaceutical University, Guangzhou 510006, China; riguangbaihe1990@126.com (L.Z.); wangfeifei351@163.com (F.W.); zqing0904@163.com (Q.Z.); QiumingLiang1021@163.com (Q.L.); smwang1966@163.com (S.W.); 2 Engineering & Technology Research Center for Chinese Materia Medica Quality of the Universities of Guangdong Province, School of Traditional Chinese Medicine, Guangdong Pharmaceutical University, Guangzhou 510006, China

**Keywords:** stybenpropol A, benzoinum, HUVECs, atherosclerosis, NF-κB and caspase-9 signaling pathways

## Abstract

Inflammation is a key mediator in the progression of atherosclerosis (AS). Benzoinum, a resin secreted from the bark of *Styrax tonkinensis*, has been widely used as a form of traditional Chinese medicine in clinical settings to enhance cardiovascular function, but the active components of the resin responsible for those pharmaceutical effects remain unclear. To better clarify these components, a new phenylpropane derivative termed stybenpropol A was isolated from benzoinum and characterized via comprehensive spectra a nalysis. We further assessed how this phenylpropane derivative affected treatment of human umbilical vein endothelial cells (HUVECs) with tumor necrosis factor-α (TNF-α). Our results revealed that stybenpropol A reduced soluble intercellular cell adhesion molecule-1 (sICAM-1), soluble vascular cell adhesion molecule-1 (sVCAM-1), interleukin-8 (IL-8), and interleukin-1β (IL-1β) expression by ELISA, inhibited apoptosis, and accelerated nitric oxide (NO) release in TNF-α-treated HUVECs. We further found that stybenpropol A decreased VCAM-1, ICAM-1, Bax, and caspase-9 protein levels, and increased the protein levels of Bcl-2, IKK-β, and IκB-α. This study identified a new, natural phenylpropane derivative of benzoinum, and is the first to reveal its cytoprotective effects in the context of TNF-α-treated HUVECs via regulation of the NF-κB and caspase-9 signaling pathways.

## 1. Introduction

Atherosclerosis (AS) is a common driver of cardiovascular and cerebrovascular disease, resulting in high and rising rates of disability and mortality globally [1,2]. Inflammation participates in all phases of AS, from plaque formation to eventual rupture, progressively narrowing the coronary arteries and resulting in a range of serious clinical complications [3]. In the best-characterized model of such inflammation, the vascular–tone imbalance between endothelin-1 (ET-1) and nitric oxide (NO) in dysfunctional endothelial cells results in the increased expression of adhesion molecules and proinflammatory factors in the vascular wall [4,5,6]. These factors, in turn, drive cholesterol accumulation and monocyte extravasation across the arterial intima, subsequently promoting proinflammatory foam cell formation and driving AS. Indeed, such inflammation-induced endothelial dysfunction and its associated programmed cell death are key drivers of AS and associated disease pathology [7,8,9]. As such, inhibition of endothelial cell apoptosis and reductions in this inflammatory response represent a promising target for constraining the occurrence and development of AS.

**Figure 1:**
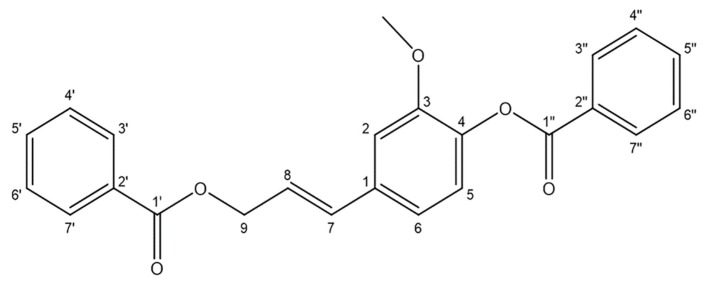
Structure of stybenpropol A.

Statins (HMG-CoA reductase inhibitors) are the primary therapeutic agents used for treating hyperlipidemia and atherosclerosis, but their clinical use is often associated with many adverse reactions such as liver damage, muscle toxicity, and gastrointestinal irritation [10,11]. Therefore, there is an urgent need to identify alternative treatments for AS. Naturally derived compounds represent a valuable source of clinically relevant compounds, with >80% of small molecule inhibitors of cancer developed from 1981–2014 having been derived from such natural compounds [12]. Benzoinum is a resin that comes from the bark of *Styrax tonkinensis* (Pier.) Craib, which is a species of tree found in many regions of Southeast Asia. Benzoinum has anti-inflammatory, antipyretic, and antitumor properties, and is clinically used in the treatment of cardiovascular diseases such as AS [13,14,15]. At present, the components of benzoinum that yield anti-AS efficacy remain unclear; thus, we explored the potential active compounds in this resin and probed their pharmacological effects and action mechanisms. In this paper, we utilized a model of tumor necrosis factor-α (TNF-α)-mediated human umbilical vein endothelial cell (HUVEC) injury in order to screen for anti-atherosclerotic compounds derived from benzoinum, obtaining a new natural product termed stybenpropol A (Figure 1) via chromatographic isolation. Stybenpropol A dramatically inhibited TNF-α-induced damage in HUVECs, potentially due to its ability to regulate the NF-κB and caspase-9 signaling pathways.

## 2. Results

### 2.1. Stybenpropol A Structural Determination

**Figure 2:**
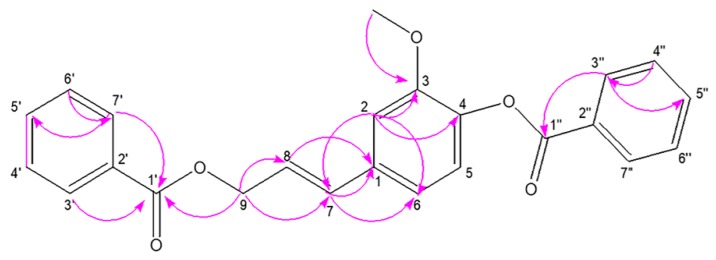
The key HMBC correlations for stybenpropol A.

Stybenpropol A (1) was obtained as a brown oil. Its molecular formula (C_24_H_20_O_5_) was determined based upon positive HR-ESI–MS at *m*/*z* 411.12003 [M + Na]^+^ (calculated for C_24_H_20_O_5_Na as 411.12029) (Figure S5), corresponding to 14 degrees of unsaturation. The IR spectral data (Figure S6) indicated absorption bands consistent with carbonyl (1717 cm^−1^) and phenyl (1601, 1508 cm^−1^) groups. The ^1^H NMR spectral data for 1, recorded in CDCl_3_ (Table 1 and Figure S1), exhibited signals consistent with two sets of benzoyloxy [one: *δ*_H_ 8.12 (2H, m), 7.51 (2H, t, *J* = 7.8 Hz), 7.57 (1H, m); the other: *δ*_H_ 8.23 (2H, m), 7.47 (2H, d, *J* = 7.8 Hz), 7.63 (1H, m); ring C1: *δ*_H_ 7.02 ´ 2 (1H, d, *J* = 8.5 Hz), 6.75 ´ 2 (1H, d, *J* = 8.5 Hz)], a set of 4-hydroxyl-3-methoxy phenyl [*δ*_H_ 7.14 (1H, d, *J* = 8.1 Hz), 7.08 (1H, d, *J* = 1.9 Hz), 7.05 (1H, dd, *J* = 8.1, 1.9 Hz), 3.84 (3H, s)], and trans allyl [*δ*_H_ 6.75 (1H, dt, *J* = 15.8, 1.4 Hz), 6.41 (1H, dt, *J* = 15.8, 6.4 Hz), 5.01 (2H, dd, *J* = 6.3, 1.4 Hz)]. When the ^13^C NMR (Table 1 and Figure S2) and HSQC (Figure S3) data were analyzed, they revealed the presence of two ester carbonyl groups (*δ*_C_ 166.4, 164.7), 20 olefinic carbons, and one methoxy group (*δ*_C_ 56.0). Therefore, 1 was speculated to be (*E*)-3-(4-(benzoyloxy)-3-methoxy-phenyl) allyl benzoate, and given the trivial name stybenpropol A based upon these findings. This determination was further validated by comparing its spectral data with related literature [16] and key HMBC correlations (Figure 2 and Figure S4), from H-9 to C-1’, C-7, and C-8, and from H-7 to C-1. The compound represents a new natural product containing no chiral carbons.

### 2.2. Stybenpropol A Protects Against TNF-α-Mediated HUVEC Injury

**Figure 3:**
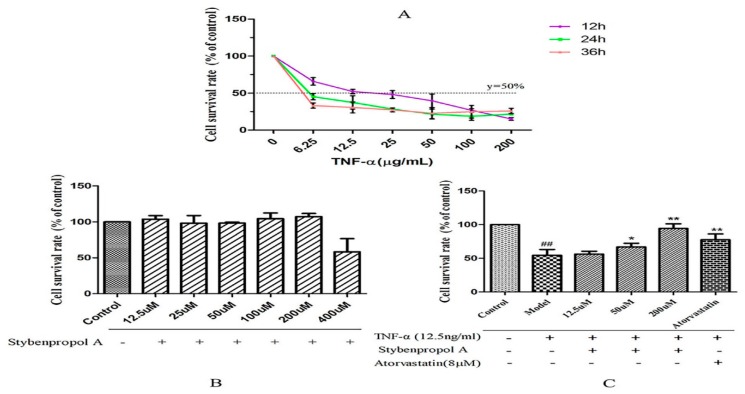
Effects of stybenpropol A on tumor necrosis factor-α (TNF-α)-mediated HUVECs. (**A**) TNF-α (6.25–200 µg/mL) was used to treat HUVECs for the indicated times (12, 24, and 36 h). A CCK-8 assay was then used to measure viability. (**B**) Stybenpropol A treatment did not decrease HUVEC viability. (**C**) HUVECs were pretreated using stybenpropol A (12.5, 50, and 200 µmol/L) or atorvastatin (8 µmol/L) for 24 h, after which TNF-α was added for 12 h and viability was assessed. Data are means ± SD of three independent experiments. ^##^ *p* < 0.01 vs. control; ∗ *p* < 0.05, ∗∗ *p* < 0.01 vs. model group.

We first constructed a model system of TNF-α-induced HUVEC injury using a range of TNF-α concentrations (0–200 ng/mL), measuring cell viability via a CCK-8 assay after 12–48 h. TNF-α mediated both time- and dose-dependent reductions in HUVEC viability relative to untreated control cells, with a 12 h treatment with 12.5 ng/mL of TNF-α leading to roughly 50% reduction in viability [17] (Figure 3A). To better explore the impact of stybenpropol A on HUVECs, we first assessed the impact of this compound on HUVEC viability. We did not detect any changes in HUVEC viability when the cells were treated for 24 h with 0–200 μM stybenpropol A (Figure 3B). We next pretreated the cells with stybenpropol A (0–200 μM) for 24 h prior to adding TNF-α for 12 h, with atorvastatin calcium serving as a positive control compound. Through this approach, we found that stybenpropol A mediated significant cytoprotection, with 200 μM being the most protective of the tested doses (Figure 3C).

### 2.3. Stybenpropol A Enhances NO Secretion in TNF-a-Treated HUVECs

**Figure 4:**
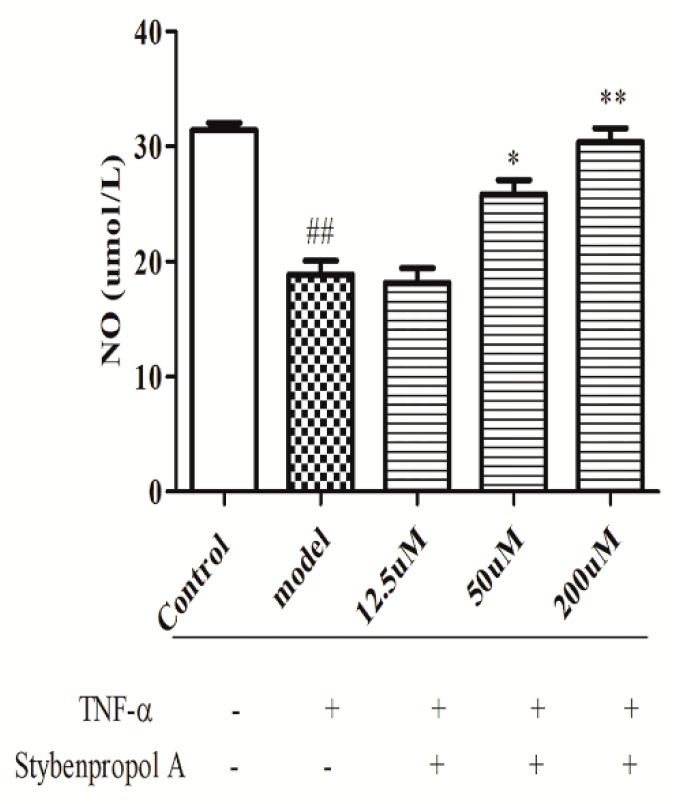
Effects of stybenpropol A on NO secretion in HUVECs treated by TNF-α. Following a 24-h pretreatment with stybenpropol A (12.5, 50, 200 μM), HUVECs were treated with TNF-α and levels of NO was measured via a NO kit. Data are means ± SD of three independent experiments. ^##^ *p* < 0.01 vs. control; ∗ *p* < 0.05, ∗∗ *p* < 0.01 vs. model group.

We next explored the relative and time-dependent effects of stybenpropol A on endothelial dysfunction by assessing HUVEC NO release. As shown in Figure 4, compared with the control cells, TNF-α-treated HUVECs have significantly reduced NO levels (*p* < 0.01). Relative to the TNF-α-treated HUVEC group, stybenpropol A had no effect at a low dose, whereas medium and high stybenpropol A doses were associated with increased NO levels in a dose-dependent fashion (*p* < 0.01 or *p* < 0.05).

### 2.4. Stybenpropol A Attenuates TNF-α-Induced Inflammation

**Figure 5:**
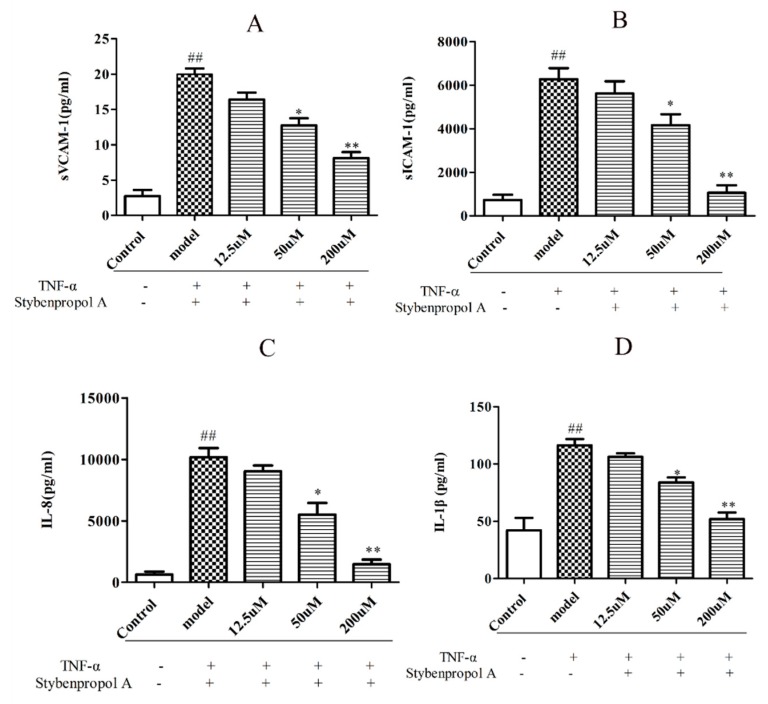
Stybenpropol A suppressed inflammation induced by TNF-α in HUVECs. Following a 24 h pretreatment with stybenpropol A (12.5, 50, 200 μM), HUVECs were treated with TNF-α for 12 h and levels of sVCAM-1(**A**), sICAM-1(**B**), IL-1β(**C**), and IL-8 (**D**) were measured via ELISA. Data are means ± SD of three independent experiments. ^##^ *p* < 0.01 vs. control; ∗ *p* < 0.05, ∗∗ *p* < 0.01 vs. model group.

To further explore the mechanisms whereby stybenpropol A may mediate anti-AS efficacy, we next assessed the impact of stybenpropol A on a soluble vascular cell adhesion molecule-1 (sVCAM-1), soluble intercellular cell adhesion molecule-1 (sICAM-1), interleukin-1β (IL-1β), and interleukin-8 (IL-8) secretion, via ELISA, after a 12 h treatment with TNF-α. We found that TNF-α treatment markedly increased the secretions of sVCAM-1, sICAM-1, IL-1β, and IL-8 by HUVECs, whereas the stybenpropol A pretreatment significantly reduced this upregulation (Figure 5A–D). Thus, these results indicated that stybenpropol A protected against the TNF-α-mediated inflammation that occurs in HUVECs.

### 2.5. Stybenpropol A Reduces TNF-α-Induced HUVEC Apoptosis

**Figure 6:**
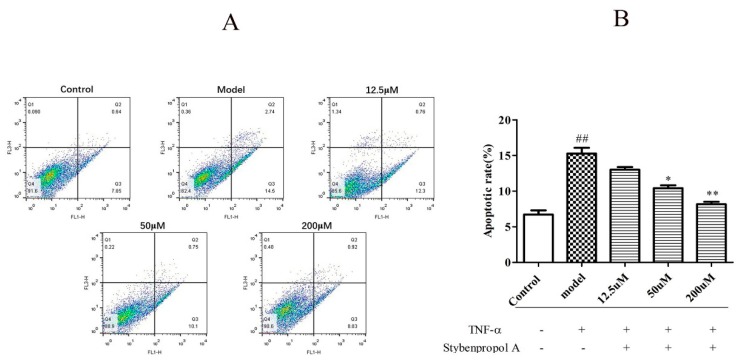
Stybenpropol A inhibited HUVEC apoptosis induced by TNF-α. Following a 24 h pretreatment with stybenpropol A (12.5, 50, 200 μM), HUVECs were treated with TNF-α and annexin V/PI staining was used to assess apoptosis (**A**). Quantification of apoptosis rates (**B**). Data are means ± SD of three independent experiments. ^##^ *p* < 0.01 vs. control; ∗ *p* < 0.05, ∗∗ *p* < 0.01 vs. model group.

We next explored how stybenpropol A (0–200 μM) affected TNF-α-induced HUVECs apoptosis. As shown in Figure 6B, relative to the control group (6.70% ± 0.83%), the frequency of apoptotic cells in the TNF-α model group significantly increased to 15.27% ± 1.16% (*p* < 0.01), indicating that TNF-α induced HUVEC apoptosis. As expected, stybenpropol A significantly reduced this TNF-α-induced apoptosis in a dose-dependent fashion. There were significant differences (*p* < 0.01 and *p* < 0.05) between the experimental and TNF-α model groups. Thus, stybenpropol A exhibited clear cytoprotective effects against TNF-α-injury in HUVECs.

### 2.6. Stybenpropol A Modulates Apoptosis-Related Protein Expression and Downregulates NF-κB Nuclear Transcription in TNF-α-Treated HUVECs

**Figure 7:**
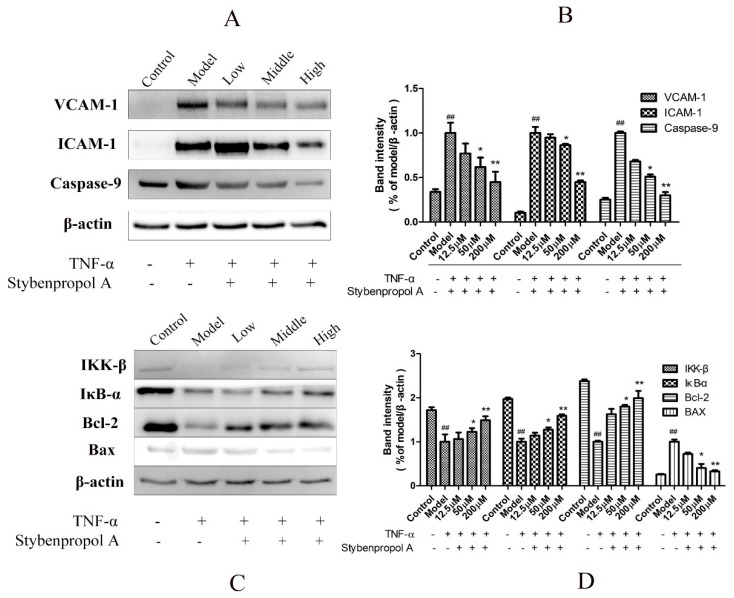
Stybenpropol A modulated apoptosis-related protein expression and downregulated NF-κB nuclear transcription. Following a 24-h pretreatment with stybenpropol A (12.5, 50, 200 μM), HUVECs were treated with TNF-α and then Western blotting was used to measure protein levels (**A**,**B**). Quantification of protein levels (**C**,**D**). Data are means ± SD of three independent experiments. ^##^ *p* < 0.01 vs. control; ^∗^ *p* < 0.05, ^∗∗^ *p* < 0.01 vs. model group.

NF-κB is a key mediator of inflammatory responses to diverse stimuli, potentially leading to the apoptotic death of cells in which it is activated [18]. We therefore assessed IκBα levels in order to gauge NF-κB activation status in this study. To fully explore the mechanisms whereby stybenpropol A mediates its anti-apoptotic efficacy, we actually assessed IKK-β, IκB-α, VCAM-1, and ICAM-1 protein levels via Western blotting. As shown in Figure 7, we found that TNF-α treatment resulted in IKK-β and IκB-α degradation in HUVECs as well as elevated VCAM-1 and ICAM-1 levels, whereas stybenpropol A reversed these effects, suggesting that stybenpropol A inhibited TNF-α-induced NF-κB activation and nuclear translocation. As such, the anti-inflammatory properties of stybenpropol A are likely linked to its regulation of NF-κB.

To further explore how stybenpropol A affects TNF-α-induced apoptosis, we assessed the expression of the apoptosis-associated Bax, Bcl-2, and caspase-9 proteins in HUVECs [19]. We found that TNF-α treatment resulted in Bcl-2 downregulation and increased Bax expression, whereas stybenpropol A reversed this effect, thereby increasing the Bcl-2/Bax ratio. Furthermore, TNF-α treatment enhanced the levels of caspase-9, and this too was inhibited by stybenpropol A [20]. These findings together suggested that stybenpropol A can enhance the expression of anti-apoptotic proteins, thus protecting against HUVEC apoptosis.

## 3. Discussion

Although once attributed to lipid deposition, AS is now thought to mainly be driven by inflammation and associated vascular endothelial dysfunction (ED) that leads to lesion development [21,22,23]. TNF-α is a potent, proinflammatory cytokine that can mediate a variety of inflammatory reactions and participate in apoptosis, autophagy, and related processes [24]. In this study, we provided novel evidence suggesting that stybenpropol A may protect TNF-α-stimulated HUVECs against inflammation and apoptosis, suppress the release of proinflammatory factors and soluble cell adhesion molecules, and promote the synthesis of NO by regulating the NF-κB and caspase-9 signaling pathways.

In AS, the endothelium exerts many vasoprotective effects that are largely mediated by NO. As endothelial NO produced by endothelial NO synthase (eNOS) exerts multiple anti-atherosclerotic effects, vascular endothelial injury and dysfunction are characterized by decreased levels of NO in endothelial cells [25,26,27]. We found that stybenpropol A increased the synthesis of NO of TNF-α-stimulated HUVEC cells, thereby potentially increasing vasodilation and protecting the vascular endothelium. The chronic expression of adhesion molecules and proinflammatory cytokines, as a consequence of ongoing inflammation, can drive monocyte adhesion and endothelial infiltration, resulting in AS development. Adhesion molecules and proinflammatory cytokines are important predictors of AS lesion development and future cardiovascular events [28]. As important mediators of vascular inflammation, these molecules are upregulated when cellular dysfunction occurs. In this study, we found that stybenpropol A significantly reduced the expression of VCAM-1, ICAM-1 IL-1β, and IL-8, in TNF-α-treated HUVECs, demonstrating that stybenpropol A can attenuate inflammation, thereby leading to a decrease in HUVEC apoptosis [1,29]. Taken together, these results support the notion demonstrating that stybenpropol A protects against TNF-α-mediated inflammatory injury in HUVECs.

To further explore the mechanisms whereby stybenpropol A acts, we explored its relevant signaling pathways in AS. NF-κB is a key mediator of AS, regulating inflammatory processes and associated cytokine expression [30,31,32]. Inactive NF-κB remains in the cytoplasm and is bound to IκB. Upon TNF-α stimulation, IκB and IKK-β are phosphorylated and rapidly degraded, releasing NF-κB, which then undergoes nuclear translocation and subsequent gene regulation [33]. As such, reduced IκB and IKK-β levels correspond to increased NF-κB activity. After the administration of stybenpropol A, ICAM-1 and VCAM-1 protein expression in TNF-α-treated HUVECs significantly decreased, while IκB-α and IKK-β levels significantly increased. This suggested that stybenpropol A may protect HUVECs against injury, associated monocyte migration, and adhesion, by suppressing the NF-κB pathway.

One key mechanism whereby inflammation is thought to drive AS is that it triggers endothelial cell apoptosis [29]. In our study, we found that stybenpropol A was able to protect TNF-α-stimulated HUVECs against apoptotic death. Caspase-9 is a key protease regulating the endogenous and exogenous pathways of apoptosis. The inhibition of its activation can block the apoptotic cascade [34]. We found that caspase-9 levels significantly increased in TNF-α-treated HUVECs, while these levels significantly decreased after pretreatment with stybenpropol A. These results indicate that stybenpropol A may inhibit the apoptosis of HUVECs by regulating caspase-9 and improving responses to inflammatory stimuli. Bax and Bcl-2 are key factors controlling the apoptotic death of HUVECs [35]. Bcl-2 inhibits apoptosis by blocking the activation of caspases, while Bax, as an important component of the mitochondrial membrane ion channel, promotes cytochrome C release from the mitochondria, activating caspase-9-mediated apoptosis [35]. We found that TNF-α upregulated caspase-9 levels and altered the ratio of Bax to Bcl-2, thereby engaging in the mitochondrial apoptosis pathway. This suggested that stybenpropol A pretreatment enhanced levels of anti-apoptotic proteins and simultaneously decreased pro-apoptotic protein levels. Together, these results show that stybenpropol A protects HUVECs from TNF-α-induced apoptosis via suppression of this mitochondrial apoptosis pathway. Collectively, these results clearly indicate that the protective effects of stybenpropol A are mediated by its suppression of the NF-κB and caspase-9 signaling pathways.

## 4. Materials and Methods

### 4.1. Reagents

The resin of *Styrax tonkinensis* (benzoinum) was purchased from Bo Zhou, Anhui Province. Stybenpropol A was isolated from benzoinum previously extracted using 95% EtOH, and its structure was identified via NMR and mass spectrometry. Compound purity was found to be >98% by HPLC. Human recombinant TNF-α was purchased from Peprotech (Princeton, NJ, USA); ECM (including HUVECs basal medium, bovine endothelial cell growth supplement (ECGS) growth factor, penicillin, and streptomycin) was obtained from ScienCell Biotechnology (Carlsbad, CA, USA); 0.25% trypsin, fetal bovine serum (FBS), and PBS, were purchased from Thermo Fisher Scientific (Waltham, MA, USA). The CCK-8 reagents were provided by Japan Tongren Company (Dojindo, Japan). The NO kits were purchased from Nanjing Institute of Bioengineering. The VCAM-1, ICAM-1, IL-Iβ, and IL-8 ELISA kits were supplied by Wuhan Cloud-Clone CORP (Wuhan, China). VCAM-1 mouse antibody, ICAM-1 rabbit antibody, IKK-β rabbit antibody, IκB-α rabbit antibody, Bax mouse antibody, Bcl-2 mouse antibody, and caspase-9 rabbit antibody were purchased from Proteintech (Sanying, Wuhan, China). The annexin-FITC apoptosis detection kit was from Beyotime (Shanghai, China). RIPA was from BestBio (Beijing, China).

### 4.2. Preparation of Stybenpropol A

After 95% ethanol extract of benzoin resin was extracted by ethyl acetate, stybenpropol A was separated by traditional column chromatography. The structure of the compound was identified by various spectral methods, utilizing ^1^H NMR, ^13^C NMR, ^1^H–^1^H COSY, HSQC, HMBC, MS, IR, and UV.

### 4.3. Cell Culture and Treatment

The HUVECs were grown in ECM basal medium supplemented with 10% FBS and 1% ECGS. The cells were cultured in a 37 °C incubator with 5% CO_2_ and were passaged 3–5 times prior to use in experiments [36]. When the cells were grown to 90% confluence, they were inoculated in 96-and 6-well plates. The experimental cells were divided into three groups: a blank control group, a model group (treated with 12.5 ng/mL TNF-α for 12 h), and an experimental group (treated with 12.5, 50, or 200 μM stybenpropol A for 24 h prior to incubation with 12.5 ng/mL TNF-α for 12 h).

### 4.4. Cell Viability

The effects of TNF-α and stybenpropol A on HUVEC viability and proliferation were assessed via plating 1 × 10^4^ cells per well of a 96-well plate in a 100 μL volume. After 24 h, the cells were treated with ECM basal medium with and without stybenpropol A (0–400 μM) for another 24 h. Then, the viability of each group was detected via the CCK-8 approach [36] with a microplate reader at 450 nm. In other experiments, after seeding in 96-well plates, the HUVECs were pre-incubated with stybenpropol A (12.5, 50, 200 μM) for 24 h, and were then treated with 12.5 ng/mL TNF-α for 12 h. To assess the protective effects of stybenpropol A on TNF-α-treated endothelial cells, cell viability was measured via the CCK-8 approach.

### 4.5. NO Content Determination

The cells were seeded in a 2 mL volume of media in 6-well plates with or without stybenpropol A (12.5, 50, 200 μM) for 24 h, after which 12.5 ng/mL TNF-α was added at 37 °C for 12 h. The collected supernatants were then assayed for NO levels according to instructions provided with an NO kit, and the maximum absorbance at 450 nm was measured via spectrophotometer [5].

### 4.6. ELISAs

The cells were seeded in a 2 mL volume of media in six-well plates with and without stybenpropol A (12.5, 50, 200 μM) for 24 h, after which 12.5 ng/mL TNF-α was added at 37 °C for 12 h. The collected supernatants were assayed for VCAM-1, ICAM-1, IL-8, and IL-Iβ levels using appropriate ELISA kits, with absorbance at 450 nm measured via microplate reader [37,38].

### 4.7. Flow Cytometry Detection of Apoptosis

An annexin V-FITC/PI apoptosis kit was used to assess HUVEC apoptosis via flow cytometry. The cells were cultured with stybenpropol A (12.5, 50, 200 μM) for 24 h before washing twice in PBS. After washing, 1 × 10^6^ cells/well were stained in a 200 µL volume of annexin-FITC binding solution that contained 5 μL of annexin-FITC. Next, 10 μL PI was added, and the cells were incubated for 20 min at room temperature (20–25 °C) in the dark followed by a flow cytometry analysis (BD FACS Verse). FlowJo was used to analyze the resultant data [39].

### 4.8. Western Blot Analysis

After a 24-h treatment with and without stybenpropol A (12.5, 50, 200 μM), the HUVECs were washed twice and then lysed with RIPA based on provided directions, after which a Bradford Protein Assay Kit (Bio-Rad, San Francisco, CA, USA) was used to quantify the protein levels in collected samples. Equal protein amounts were then separated through 10% SDS-PAGE, and transferred to PVDF membranes (Millipore, Danvers, MA, USA), which were blocked for one h at room temperature with 5% non-fat milk. The blots were then probed using primary anti-β-actin, anti-VCAM-1, anti-ICAM-1, anti-Bax, anti-caspase-9, anti-Bcl-2, anti-IKK-β, and anti-IκB-α antibodies (1:1000) at 4 °C overnight. Next, the blots were probed with appropriate HRP-conjugated secondary antibodies (1:5000) for 2 h at room temperature. The blots were then washed four times for 5 min per wash using TBST, followed by protein detection with a ChemiDoc™ XRS+ System (Sagecapt, Beijing, China) using an enhanced chemiluminescence (ECL) reagent [6].

### 4.9. Statistical Analysis

Data are means ± SD. SPSS 20.0 was used for statistical analyses, while GraphPad Prism 7.00 was used for figure construction. One-way ANOVAs were used for comparisons between groups, while LSD was used for pairwise comparisons. *p* < 0.05 was the significance threshold.

## 5. Conclusions

In summary, our results demonstrate that stybenpropol A, a novel phenylpropane derivative isolated from benzoinum extract, inhibits inflammatory damage, increases NO levels, reduces vascular adhesion and proinflammatory factor levels, inhibits apoptosis, and protects vascular endothelial cells from damage and death. This protective effect was linked to the inhibition of NF-κB translocation and the increased expression of anti-apoptotic Bcl-2. This suggests that stybenpropol A may represent a promising compound for preventing and treating AS, provided these results are validated in animal and clinical trials.

## Figures and Tables

**Table 1:** ^1^H (500 MHz) and ^13^C NMR (125 MHz) data of 1 in CDCl_3_ (*δ*, ppm).

Position	*δ*C	*δ*H (mult, *J* in Hz)	Position	*δ*C	*δ*H (mult, *J* in Hz)
1	135.4		2′	130.2	
2	110.4	7.08 d (1.9)	3′,7′	129.7	8.12 m
3	151.5		4′,6′	128.6	7.51 t (7.8)
4	140.0		5′	133.1	7.57 m
5	123.1	7.14 d (8.1)	1″	164.7	
6	119.5	7.05 dd (8.1,1.9)	2″	129.4	
7	133.6	6.75 dt (15.8,1.4)	3″,7″	130.4	8.23 m
8	123.7	6.41 dt (15.8,6.4)	4″,6″	128.5	7.47 t (7.8)
9	65.5	5.01 dd (6.3,1.4)	5″	133.7	7.63 m
1′	166.4		–OCH_3_	56.0	3.84 s

## Supplementary Materials

Supplementary materials can be found at https://www.mdpi.com/1422-0067/20/21/5383/s1.
