# Supplementary material for: Anti-Inflammatory and Anti-Apoptotic Effects of Stybenpropol A on Human Umbilical Vein Endothelial Cells
Source: Int J Mol Sci. 2019 Oct 29;20(21):5383. doi: 10.3390/ijms20215383 (PMC6862503; doi:10.3390/ijms20215383)
Supplement: Supplementary file 1 [file ijms-20-05383-s001.pdf]

Anti-inflammatory and Anti-apoptotic Effects of Stybenpropol A Isolated from Benzoinum on Human Umbilical Vein Endothelial Cells

Li Zhang [1,†], Feifei Wang[1,†], Qing Zhang [1], Qiuming Liang[1], Shumei Wang[1,2], Minghua Xian*[1,2], and Feng Wang*[1,2]

[1] Key Laboratory of Digital Quality Evaluation of Chinese Materia Medica of State Administration of TCM, School of Traditional Chinese Medicine, Guangdong Pharmaceutical University, Guangzhou 510006, China

[2] Engineering & Technology Research Center for Chinese Materia Medica Quality of the Universities of Guangdong Province, School of Traditional Chinese Medicine, Guangdong Pharmaceutical University, Guangzhou 510006, China.

* Correspondence: e-mail: wfeng1230@163.com; Fax: +86 020-39352174; Tel: +86 18676625933

† Authors contributed equally to this work.

Contents of Supporting Information

No.		Page
1	Extraction and isolation of **1**	2
2	Figure S1. ^{1}H NMR spectrum of **1** in MeOD.	3
3	Figure S2. ^{13}C NMR spectrum of **1** in MeOD.	3
4	Figure S3. HSQC spectrum of **1** in MeOD.	4
5	Figure S4. HMBC spectrum of **1** in MeOD.	4
6	Figure S5. HRESIMS of **1**.	5
7	Figure S6. IR spectrum of **1**.	6

Extraction and isolation of **1**

Benzoinum (5 kg) was extracted by maceration in EtOH/H_2O (95:5, 3 × 40 L) at room temperature, giving a crude extract (4.2 kg). The crude extract was suspended in water and extracted with petroleum ether and EtOAc, sequentially, and afforded an EtOAc-soluble extract (3.8 kg). The EtOAc-soluble fraction (2.0 kg) was subjected to a silica gel column using a petroleum ether/EtOAc gradient solvent system (50:1 to 0:100) to give seven fractions (B1−B7). Fraction B2 (598 g) was chromatographed over a silica gel column with petroleum ether/EtOAc mixtures (40:1 to 0:100) to yield fractions B2.1-B2.7. Fraction B2.2 (21.2 g) was fractionated into six parts (B2.2.1-B2.2.6) via MCI gel CHP 20P (MeOH/H_2O, 30:70 to100% MeOH). Fraction B2.2.4 (4.0 g) was separated by low and medium pressure ODS column eluted with MeOH/H_2O followed by semi-preparative HPLC (MeOH /H_2O, 80%) to yield **1** (6 mg).

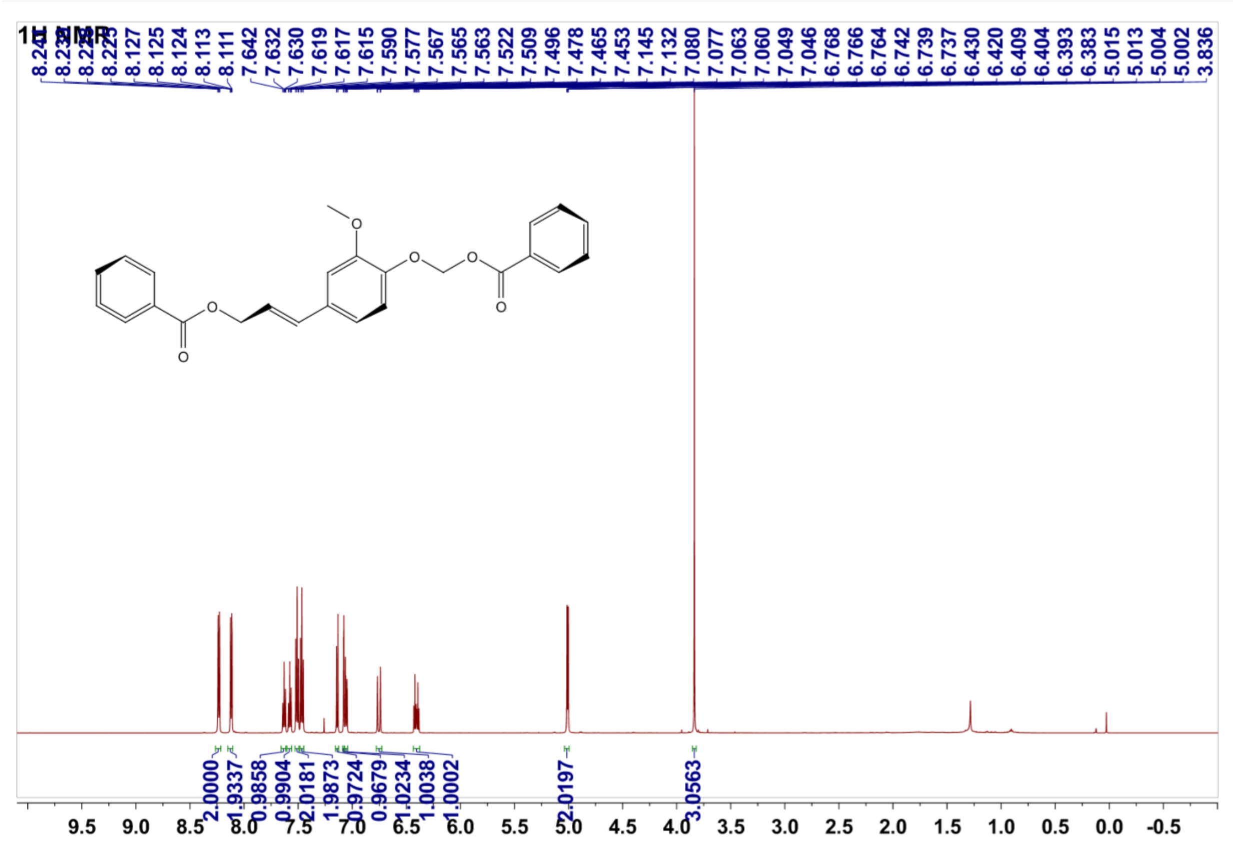

Figure S1. 1H NMR spectrum of 1

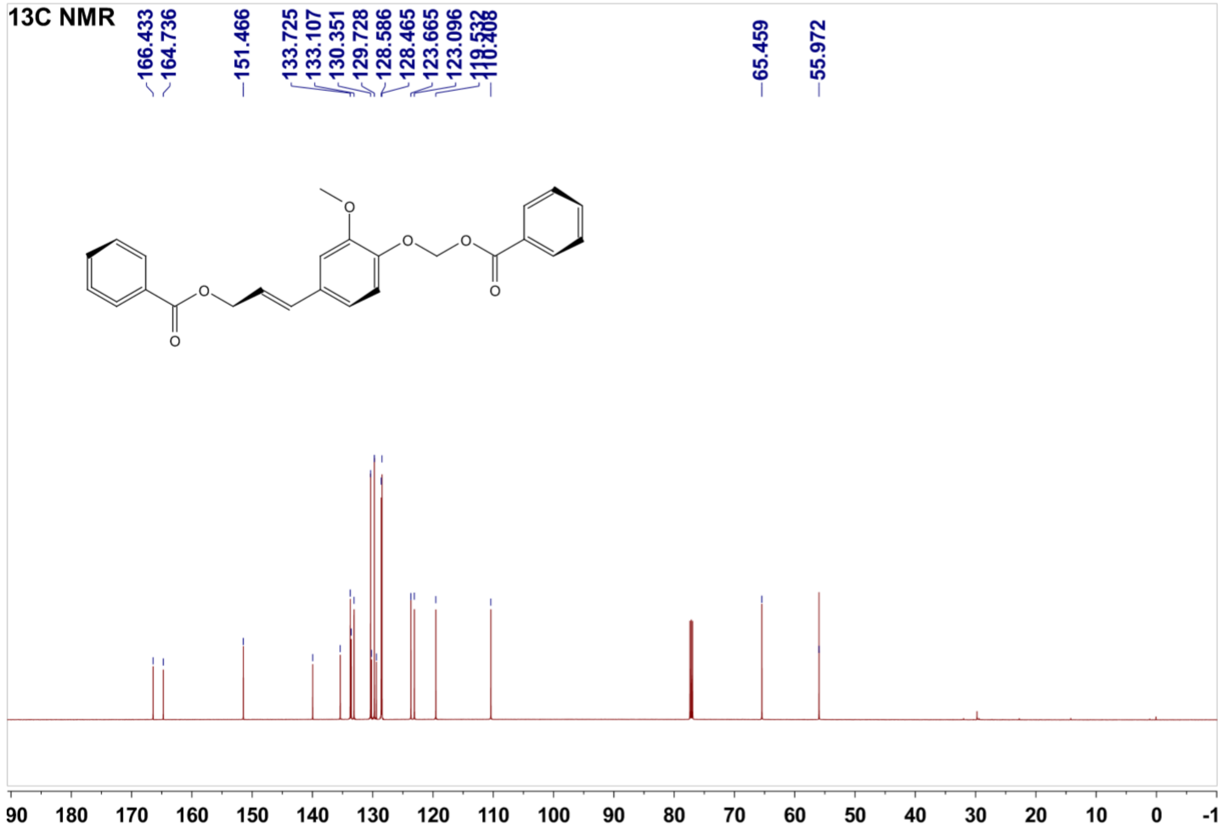

Figure S2. ^{13}C NMR spectrum of **1**

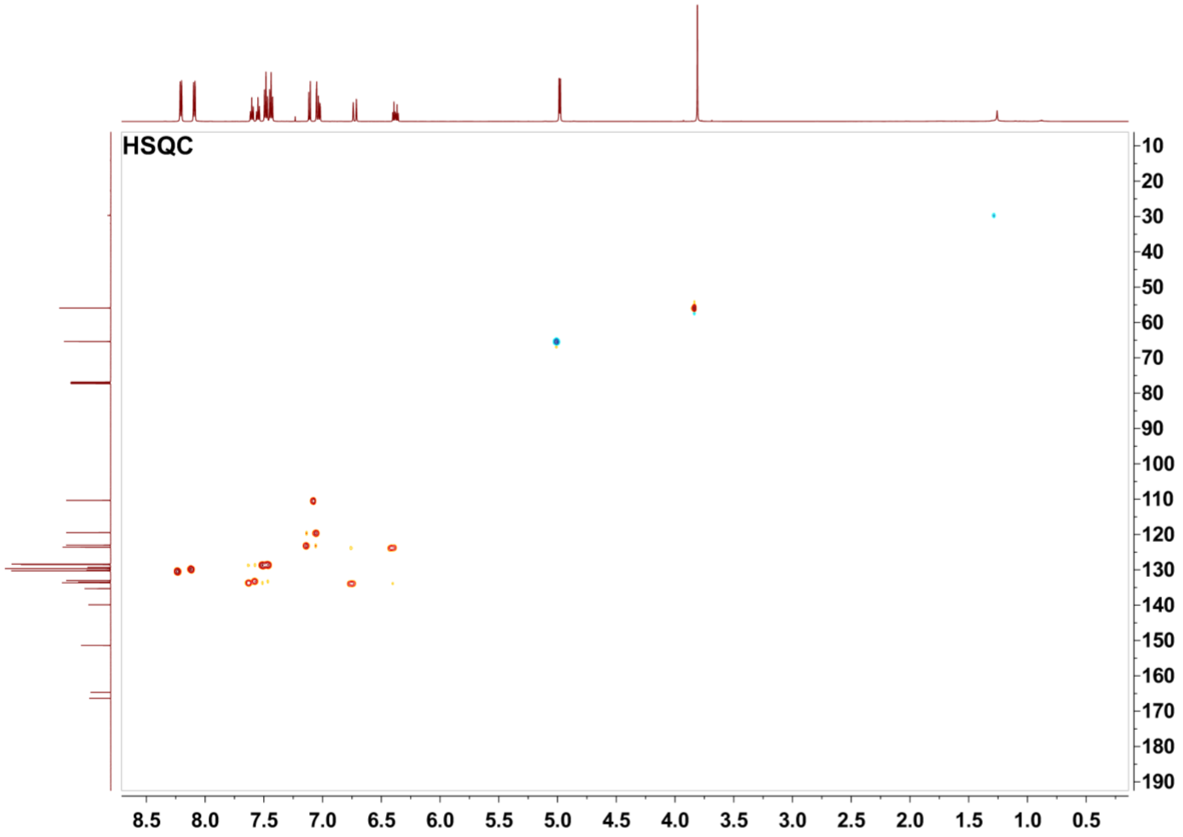

Figure S3. HSQC spectrum of **1** in MeOD.

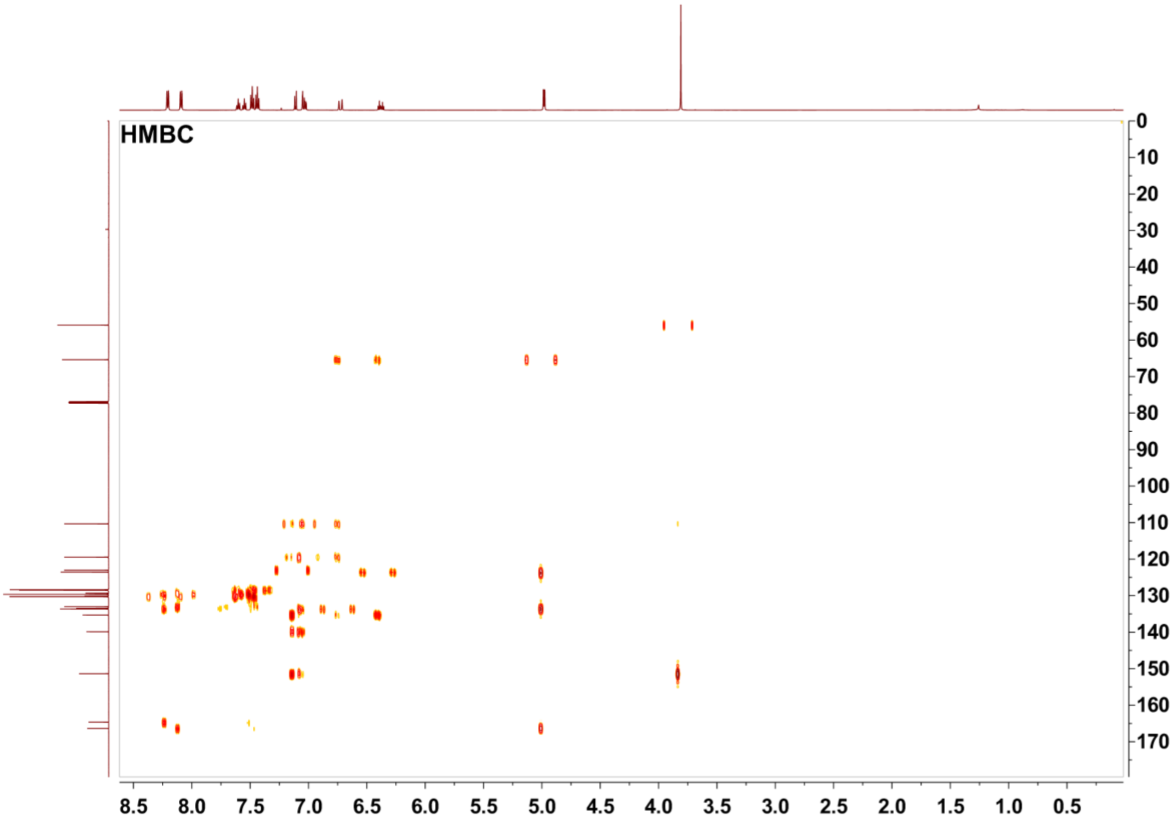

Figure S4. HMBC spectrum of **1** in MeOD.

Thermo

Mass Analysis Report

**Sample Name**	**Operator**	**Acquisition Date**	**Method**
S2-3-2-2	Q Exactive	11/22/2018	C:\Xcalibur\methods\fs_sim_ESI
**Scan Mode**	**Scan polarity**	**Resolution**	**Isolation windown（m/z）**
Full MS<br>Targeted-SIM	Negative	70000	2

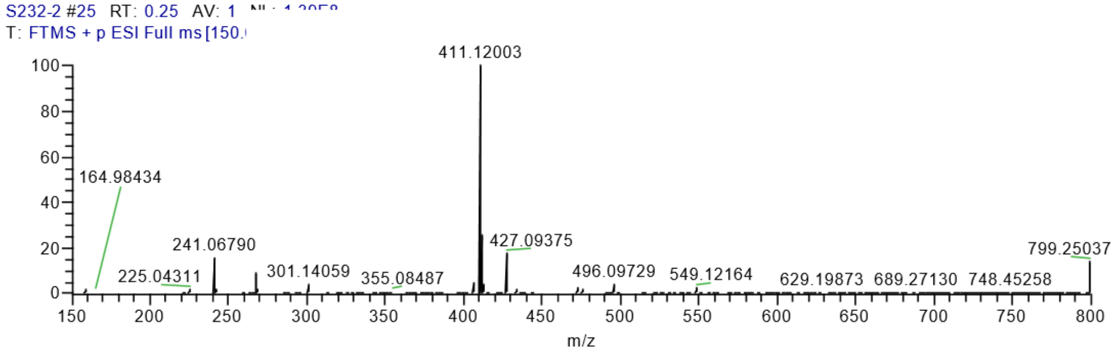

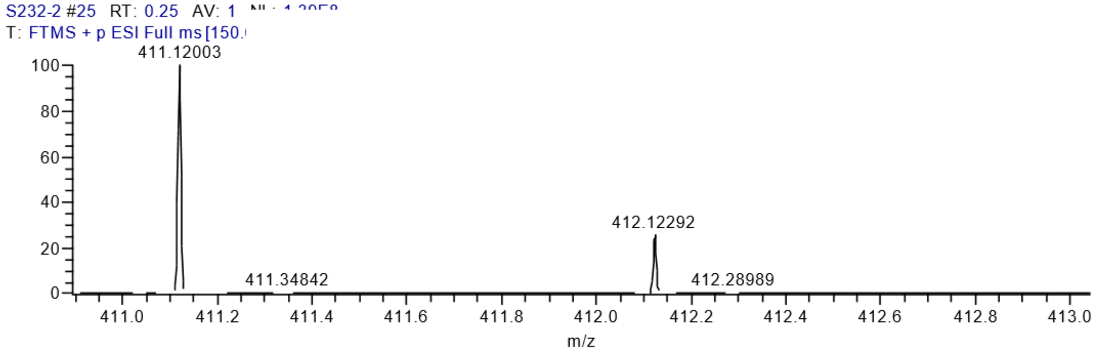

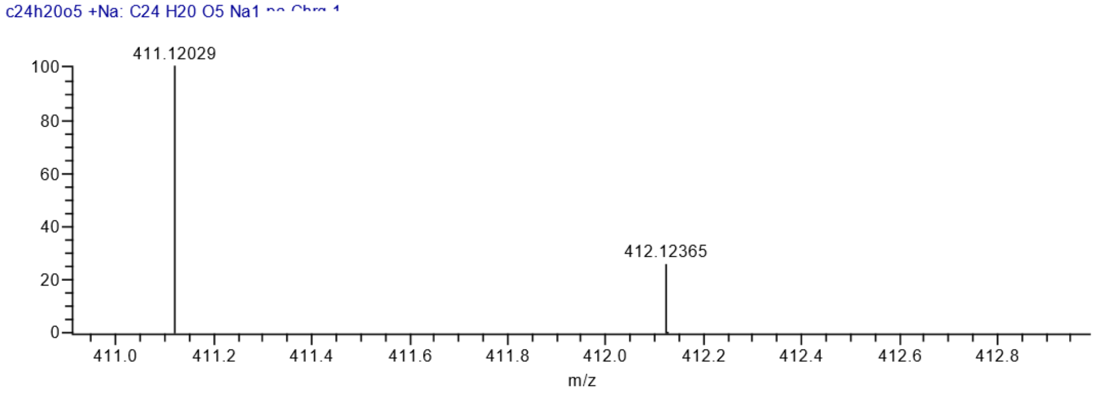

Formula	Species	Expected m/z	Measured m/z	Delta [ppm]
C24H20O5	+ Na	411.12029	411.12003	-0.63

Figure S5. HRESIMS of **1**.

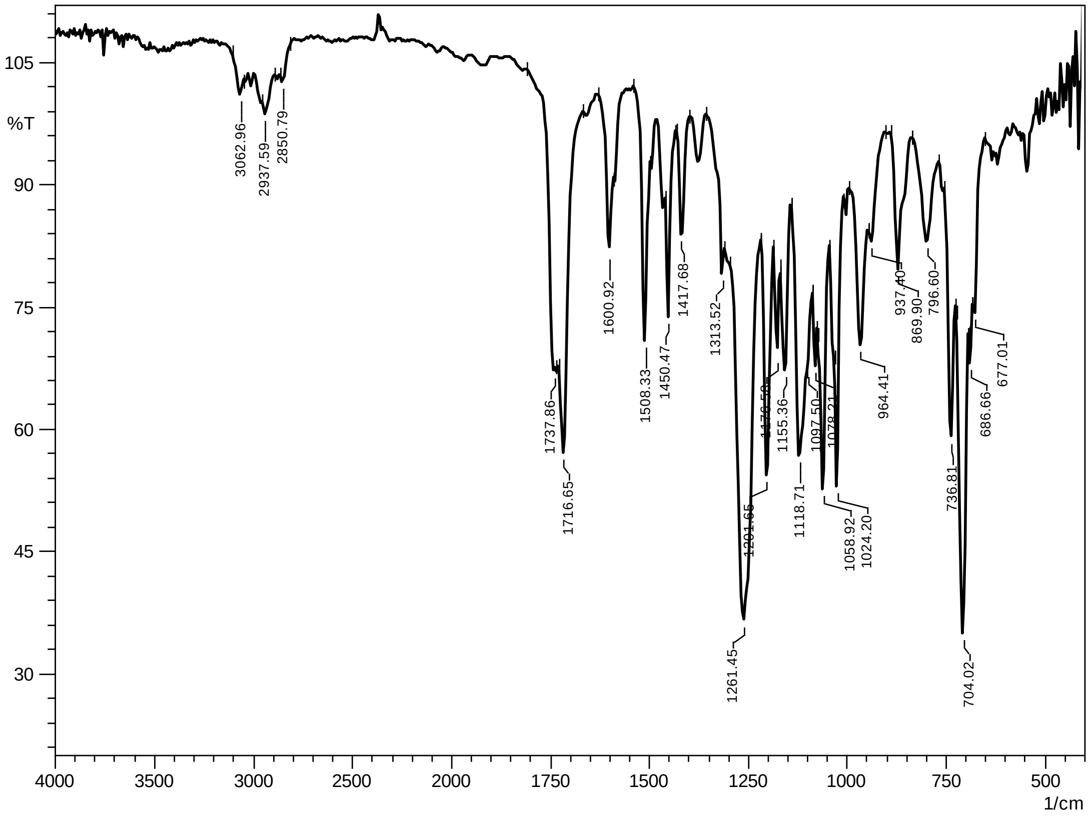

Figure S6. IR spectrum of **1**.
